# Understanding Graduate Students’ Perspectives on Food Apps to Inform User-Centered Design: Explanatory Sequential Mixed Methods Survey Study

**DOI:** 10.2196/84576

**Published:** 2026-03-27

**Authors:** Mehrnoush Mokhtarnejad, Lyda Fontes McCartin

**Affiliations:** 1 School of Information Science, University of South Carolina, 1501 Greene Street, Columbia, SC, 29208, United States, 1 803-777-0169

**Keywords:** food apps, graduate students, healthy eating, food waste, mobile apps, mixed methods, COM-B model, capability, opportunity, and motivation-behavior model

## Abstract

**Background:**

Mobile food apps have the potential to promote healthier eating behaviors and more sustainable food practices. Graduate students often struggle to maintain healthy dietary habits due to lifestyle transitions, academic stress, limited time, and constrained budgets, which can lead to poor meal planning, irregular eating patterns, and increased food waste.

**Objective:**

This study aimed to examine graduate students’ dietary behaviors, food waste practices, and preferences for mobile food app features, with the intention of informing the design of user-centered tools that promote healthy eating and reduce food waste.

**Methods:**

This study used an explanatory sequential mixed methods design. In the first phase, 63 graduate students at the University of South Carolina completed an online survey between November and December 2024, which captured demographics, cooking habits, dietary preferences, food waste behaviors, and online recipe search behaviors. Findings from the survey shaped the design of interview questions, allowing qualitative inquiry to explain and expand upon the quantitative patterns. Ten purposively selected participants completed semistructured interviews. Quantitative data were analyzed descriptively and using the chi-square test with R software (version 4.3.1), while qualitative data were transcribed and thematically analyzed.

**Results:**

Survey findings revealed that 54% (34/63) of the participants ate out daily or almost daily; 25.3% (16/63) consumed fast, frozen, or canned food at least 3 to 5 times per week; and only 19% (12/63) cooked daily. Most participants consumed only 1 serving of vegetables (43/63, 68.2%) and fruits (33/63, 52.4%) daily. Nearly 70% (44/63) reported food waste, primarily from leftovers and unused ingredients. A significant association was found between shopping list use and reduced food waste (*χ*²_1_=9.66, *P*=.008; Cramér V=0.39). The interviews contextualized these patterns: the students described time constraints, limited cooking skills, and overbuying as key barriers to healthy eating and waste reduction, while recommending app features such as nutrition tracking, batch meal planning, personalized dietary filters, ingredient-based recipe generators, and grocery list tools with reminders. Participants also emphasized the importance of simple design, multimodal content, and accurate cooking time estimates.

**Conclusions:**

This study demonstrates that integrating quantitative and qualitative insights through the capability, opportunity, motivation-behavior (COM-B) framework provides a nuanced understanding of graduate students’ dietary practices, food waste behaviors, and app feature preferences. The findings highlight the need for mobile food apps that are not only evidence based but also user centered, offering simple, time-efficient, and customizable tools that simultaneously address capability deficits, opportunity barriers, and motivation maintenance. Such apps have the potential to address barriers unique to graduate students, such as limited time, minimal cooking skills, and organizational challenges, while supporting healthier eating and reducing food waste.

## Introduction

### Background

Unhealthy eating patterns and poor lifestyle behaviors are major contributors to chronic conditions such as obesity, cardiovascular disease, diabetes, and certain cancers [1]. The World Health Organization estimates that up to 80% of cases involving cardiovascular illness, stroke, metabolic disorders, and type 2 diabetes can be prevented through healthier dietary choices [2]. Concurrently, technological innovation has created new opportunities for promoting health and sustainability [3,4]. Mobile food apps now offer features such as recipe suggestions, meal planning tools, and grocery shopping assistance designed to support informed dietary decisions and environmentally sustainable food practices [5,6]. However, the adoption and effectiveness of these apps vary widely across user groups [7], making it essential to understand how specific populations engage with these tools to design inclusive, user-centered digital health solutions.

University students face particular challenges in maintaining healthy dietary habits due to academic pressures, limited time, inconsistent schedules, minimal cooking skills, and budget constraints [8,9]. These factors often lead to irregular eating patterns, reliance on convenience foods, and increased food waste [10-12]. As individuals in a transitional life stage, students are at risk of developing long-term dietary habits that impact future health outcomes [13-16]. Furthermore, studies show that busy lifestyles further hinder leftover management and contribute to food waste, making young adults and university students among the largest contributors to avoidable food waste, yet they remain an understudied group in this context [17,18].

Research indicates that graduate students are more likely to experience food insecurity than undergraduates [19]. Nearly half of graduate students experience some level of food insecurity [20-22]. Graduate students face persistent financial insecurity, chronic stress, and professional pressures that contribute to disordered eating and low dietary quality [23-26]. These challenges stem from structural constraints including low stipends, inflexible assistantship funding, high tuition, visa restrictions limiting employment, and limited opportunities for supplemental income [27-29]. Given these unique vulnerabilities and the limited research on digital nutrition interventions tailored to this population, graduate students warrant dedicated study to develop user-centered solutions that address their specific barriers to healthy eating and food waste reduction.

To address the gap in research on mobile food apps for graduate students, this study used an explanatory sequential mixed methods design to explore their dietary behaviors, food waste practices, and preferences for app features. Survey data first identified the broad patterns and prevalence of cooking, diet, and food waste behaviors, which directly informed follow-up interview questions exploring the underlying reasons and user needs. This integration captured not only the prevalence of behaviors but also the factors driving them, ensuring that insights from both phases contributed to user-centered app design recommendations. By linking quantitative patterns with qualitative insights, this study offers guidance for developing mobile food apps that promote healthier eating and reduce food waste among graduate students.

### Theoretical Framework

This study is guided by the capability, opportunity, motivation-behavior (COM-B) model, a widely used framework for understanding and designing behavior change interventions [30]. The model posits that behavior occurs when individuals have the necessary capability (physical and psychological skills), opportunity (environmental and social factors), and motivation (reflective and automatic processes). Mobile health (mHealth) apps can address barriers across all 3 components through skills training (capability), planning tools and reminders (opportunity), and goal setting features (motivation). By mapping graduate students’ reported barriers and preferences to specific behavior change techniques (BCTs) [31], this study identified app features most likely to support sustained dietary behavior change and food waste reduction.

## Methods

### Study Design

A mixed methods study using an explanatory sequential design was conducted between November and December 2024 involving graduate students at the University of South Carolina. With this design, survey results provided a broad understanding of graduate students’ cooking habits, dietary preferences, and food waste, while interviews provided deeper explanations and contextual insights that clarified and expanded upon the quantitative findings.

In the first phase, 63 participants completed a structured online survey that collected data on demographics, cooking habits, dietary preferences, food waste behaviors, and online recipe search behaviors. These results provided a broad overview of the eating and food management practices of students and revealed patterns and trends requiring further exploration.

For the second phase, survey participants indicated their willingness to participate in a follow-up interview by providing their email address at the end of the survey. From this pool, 10 participants were purposively selected to ensure diverse perspectives across key demographic and academic characteristics. Selection criteria aimed to capture variation in gender, race, and ethnicity; academic discipline; and living arrangements. The final interview sample included 5 women, 4 men, and 1 participant identifying as another gender. Participants represented diverse racial and ethnic backgrounds: 4 White, 2 Asian, 2 Middle Eastern or North African, 1 Black or African American, and 1 Hispanic or Latino/a/x. Living arrangements varied, with 3 participants living alone, 3 living with roommates, and 4 living with a partner or married. Semistructured interviews lasting approximately 30 to 35 minutes were conducted either online or in person, according to participant preference, in a private setting. All interviews were audio-recorded and transcribed verbatim for analysis.

Quantitative and qualitative data were integrated through a systematic connecting process. This study used a connecting approach to mixed methods integration [32], in which quantitative survey results directly informed the development of qualitative interview questions, and qualitative findings were then used to explain and expand upon quantitative patterns. Integration occurred at 3 distinct stages: design, data collection, and interpretation. At the design stage, the explanatory sequential structure inherently connected the 2 phases, with phase 1 (survey) results shaping phase 2 (interview) protocol development. At the data collection stage, survey data were analyzed immediately upon collection to identify patterns requiring deeper exploration. Specific quantitative findings directly informed interview question development. At the interpretation stage, qualitative and quantitative findings were merged through a joint display approach, systematically comparing survey patterns with interview themes to create integrated interpretations.

### Participant Recruitment

Participants were recruited from the graduate student population at the University of South Carolina between November and December 2024. Graduate students registered in 4 different graduate-level courses at the University of South Carolina (total enrollment: 121 students) were invited to participate. These 4 courses enrolled graduate students from diverse disciplines across multiple graduate programs university-wide. Recruitment invitations were distributed via students’ university email addresses. The email invitation described the study’s purpose and time commitment and provided a link to the online survey (Multimedia Appendix 1).

Of the 121 graduate students, 63 completed the survey, yielding a response rate of 52.1%. For the interview phase, survey participants indicated their willingness to participate in a follow-up interview by providing their email address at the end of the survey. From this pool, 10 participants completed interviews, which were conducted either online or in person, according to participant preference. No incentives or compensation were offered for participation.

### Interview Protocol Development

The semistructured interview protocol was developed specifically for this study on the basis of patterns and associations identified in the survey data. Following the explanatory sequential design framework [33], interview questions were crafted to explain and expand upon quantitative findings. For example, survey data showing that the majority of participants consumed only 1 serving of vegetables per day informed interview questions exploring barriers to healthy cooking and vegetable consumption. Similarly, the high prevalence of food waste and the significant association between shopping list use and reduced waste shaped interview prompts about grocery shopping habits, meal planning strategies, overbuying behaviors, and leftover management. The interview guide included open-ended questions about participants’ cooking practices, dietary challenges, food waste experiences, current use of food-related technologies, and recommendations for mobile app features that would support healthier eating and waste reduction. The semistructured format allowed the interviewer to probe deeper into participants’ responses while ensuring consistency across interviews in addressing key topics derived from the survey phase.

### Inclusion and Exclusion Criteria

Participants were required to meet the following inclusion criteria: (1) currently enrolled as a graduate student (master’s or doctoral level) at the University of South Carolina during the 2024 fall semester and (2) at least 18 years of age. No exclusion criteria were applied on the basis of degree program, nationality, dietary restrictions, or food security status. Food insecurity was not assessed as part of the screening or inclusion criteria, as the study aimed to capture a broad spectrum of graduate students’ dietary behaviors and food waste practices regardless of food security status. While food insecurity may moderate the diet quality and waste behaviors, this study focused on understanding the general patterns, barriers, and app feature preferences across the graduate student population to inform inclusive app design that could benefit students with varying levels of food access and financial resources.

### Ethical Considerations

This study was approved by the University of South Carolina Institutional Review Board (Pro00141073). All participants received an information sheet outlining the study’s purpose, procedures, potential risks, and confidentiality protocols prior to participation. Informed consent was obtained from all participants before they completed the survey and again before they participated in interviews. Participation was entirely voluntary, and participants were informed of their right to withdraw at any time without penalty. All data were anonymized to protect participant privacy, with no personally identifiable information linked to survey responses or interview transcripts. Participants were not compensated for their participation in this study.

### Data Analysis

Quantitative data were analyzed descriptively using frequencies and percentages to characterize participants’ demographics, dietary behaviors, and food waste practices. Inferential analysis was performed using the chi-square test of independence to examine the association between categorical variables, specifically shopping list use and food waste occurrence. Statistical analyses were conducted using R software (version 4.3.1; R Foundation for Statistical Computing). Statistical significance was set at *α*=.05 (2-tailed). Effect sizes were calculated using Cramér V to assess the practical significance of observed associations. Given the exploratory nature of the quantitative phase, only 1 inferential test was conducted to avoid multiple comparison issues.

Qualitative data were analyzed using thematic analysis following the approach of Braun and Clarke [34]. Analysis was conducted manually using Microsoft Excel for data management and organization. The lead researcher conducted the initial coding by reading all transcripts multiple times; highlighting relevant text segments; and assigning preliminary codes inductively on the basis of content related to barriers, preferences, and app feature recommendations. Coded segments were systematically organized in Excel, allowing for iterative refinement and categorization of codes into broader themes. To enhance credibility and confirmability, the second author independently reviewed the coded transcripts and the emerging codebook. Discrepancies were resolved through discussion until consensus was reached on code definitions and theme structure. The lead researcher maintained analytic memos throughout the coding process, documenting coding decisions, emerging patterns, and reflexive notes about potential interpretive biases. Final themes were identified through iterative comparison across transcripts, with attention to both convergent patterns and divergent perspectives among participants.

A total of 63 graduate students participated in the study (Table 1). Most of the 63 participants were female students (n=33, 52.4%) and aged between 22 and 32 years (n=45, 71.4%). Participants represented diverse racial and ethnic backgrounds, with the largest groups identifying as White (n=21, 33.3%), Asian (n=14, 22.2%), and Middle Eastern or North African (n=14, 22.2%). Over half (n=39, 61.9%) of the participants used iOS devices. Regarding living arrangements, the majority lived with roommates (n=32, 50.8%) or with family or partners (n=19, 30.2%), while some lived alone (n=12, 19%). This diversity across age, gender, ethnicity, and living situation provides important information for interpreting their food habits and app preferences.

**Table 1.** Demographic characteristics of the participants (N=63).

Category	Participants, n (%)
Gender	
Female	33 (52.4)
Male	25 (39.6)
Other	5 (8)
Age range, years	
22‐32	45 (71.4)
33‐44	16 (25.4)
45‐55	2 (3.2)
Race and ethnicity	
White (non-Hispanic)	21 (33.3)
Black or African American (non-Hispanic)	11 (17.5)
Hispanic or Latino/a/x	2 (3.2)
Asian	14 (22.2)
Middle Eastern or North African	14 (22.2)
Multiracial/other	1 (1.6)
Smartphone operating system	
Android	24 (38.1)
iOS	39 (61.9)
Current living arrangement	
Living alone	12 (19)
Living with roommates	32 (50.8)
Living with partner or family	19 (30.2)

## Results

In this section, we present the quantitative survey findings on graduate students’ dietary behaviors, food choices, meal-related habits, grocery shopping practices, and self-reported food waste experiences, followed by key qualitative themes from the semistructured interviews. Table 2 shows the distribution of dietary behaviors, food choices, and meal-related habits among participants.

Table 2 shows that while nearly half of the 63 participants (n=30, 47.6%) cooked a few times a week, only 19% (n=12) cooked daily, and 14.3% (n=9) engaged in weekly meal preparation. Nutritional value influenced food choices for the majority of participants, with 85.7% (n=54) indicating that it mattered at least somewhat. Fast, frozen, or canned food was consumed a few times a month (n=24, 38.1%) or 1 to 2 times per week (n=20, 31.7%). Fast, frozen, and canned food was grouped as a single survey item to capture the overall reliance on convenience foods. While nutritionally distinct, these options share the common characteristic of requiring minimal preparation time, making them appealing choices for time-constrained graduate students.

Most participants consumed only 1 serving of vegetables (n=43, 68.2%) and fruit (n=33, 52.4%) daily, with 28.5% (n=18) reporting no daily fruit intake. Eating out was frequent, with 54% (n=34) doing so daily or almost daily. In addition, many students regularly searched for food recipes online, with 20.6% (n=13) always doing so, 44.5% (n=28) sometimes, 33.3% (n=21) rarely, and only 1.6% (n=1) never.

**Table 2.** Dietary behaviors and food choices among participants (N=63).

Category	Participants, n (%)
Cooking frequency	
Daily	12 (19)
A few times a week	30 (47.6)
A few times a month	10 (15.9)
Meal preparation for the entire week	9 (14.3)
Rarely or never	2 (3.2)
Impact of nutritional value on food choices	
A great deal	27 (42.9)
Somewhat	27 (42.9)
Very little	9 (14.2)
Frequency of fast, frozen, or canned food consumption	
A few times a month	24 (38.1)
1‐2 times a week	20 (31.7)
3‐5 times a week	12 (19)
Every day	4 (6.3)
Never	3 (4.8)
Daily vegetable servings consumed	
1 serving	43 (68.2)
2 servings	9 (14.3)
≥3 servings	3 (4.8)
None	8 (12.7)
Daily fruit servings consumed	
1 serving	33 (52.4)
2 servings	10 (15.9)
≥3 servings	2 (3.2)
None	18 (28.5)
Frequency of eating out among participants	
Daily or almost daily	34 (54)
3‐5 times a week	17 (27)
1‐2 times a week	10 (15.9)
1‐2 times a month	1 (1.5)
Never	1 (1.5)
Frequency of searching for food recipes online	
Always	13 (20.6)
Sometimes	28 (44.5)
Rarely	21 (33.3)
Never	1 (1.6)

**Figure 1.**
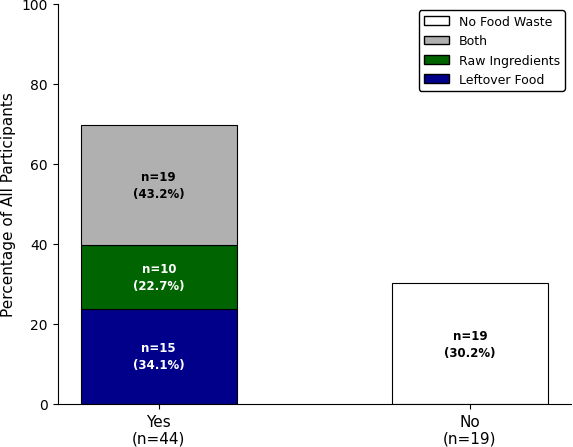
Prevalence and primary source of food waste among participants (N=63).

Food waste was assessed by asking participants the following question: “Do you experience food waste? (Yes/No)”. Participants who responded “Yes” were then asked to identify the primary source(s) of their food waste: leftover food, unused raw ingredients, or both. The measure reflects students’ self-assessed experience with food waste rather than objective waste volumes or rates. Figure 1 shows that of the 63 participants, 44 (69.8%) reported experiencing food waste, while 19 (30.2%) indicated that they did not. Of the 44 participants who experienced food waste, 15 (34.1%) cited leftover food, 10 (22.7%) mentioned raw ingredients, and 19 (43.2%) reported both as primary sources. In the interview phase, food waste was explored in depth through follow-up questions that provided rich contextual detail about what the participants waste, why, and when.

Table 3 shows that most of the 63 participants shopped weekly (n=39, 61.9%), with others shopping every 2 weeks (n=18, 28.6%), monthly (n=5, 7.9%), or daily (n=1, 1.6%). When asked about meal planning before shopping, over half (n=33, 52.4%) stated that they sometimes planned meals, while 30.1% (n=19) always planned meals. A majority (n=38, 60.3%) reported using a shopping list during grocery trips.

A chi-square test of independence revealed a statistically significant association between using a shopping list and experiencing food waste (*χ*²_1_=9.66, *P*=.008; Cramér V=0.39). Table 4 presents the contingency table showing this relationship. Among participants who did not use shopping lists, 92% (23/25) reported food waste compared to 55.3% (21/38) of those who used shopping lists. The medium to large effect size (V=0.39) indicates a strong association between structured planning through shopping list use and reduced food waste among graduate students.

**Table 3.** Grocery shopping behaviors and meal planning practices among participants (N=63).

Category	Participants, n (%)
How often do you go grocery shopping?	
Daily	1 (1.6)
Weekly	39 (61.9)
Every 2 weeks	18 (28.6)
Monthly	5 (7.9)
Do you plan meals before going grocery shopping?	
Always	19 (30.1)
Sometimes	33 (52.4)
Rarely	7 (11.1)
Never	4 (6.3)
Do you use a shopping list when you go grocery shopping?	
Yes	38 (60.3)
No	25 (39.7)

**Table 4.** Association between shopping list use and food waste among participants (N=63).

	Food waste: yes	Food waste: no	Total
Uses shopping list, n (%)	21 (55.3)	17 (44.7)	38 (100)
Does not use shopping list, n (%)	23 (92)	2 (8)	25 (100)
Total, n (%)	44 (69.8)	19 (30.2)	63 (100)

### Interview Findings

The semistructured interview protocol was informed by survey findings, particularly items related to cooking habits, dietary behaviors, food waste, and grocery shopping practices. For example, survey data showing that a majority of participants consumed only 1 serving of vegetables per day informed the interview questions about barriers to healthier eating. Similarly, reported challenges with food waste guided interview prompts about food waste behavior and strategies to reduce waste. Also, survey results showed a statistically significant association between using a shopping list and experiencing food waste. This quantitative finding directly shaped the interview questions about grocery shopping habits, planning strategies, and app features promoting waste minimization.

### Overview

Based on the interview data, three overarching themes emerged from qualitative interviews: (1) suggestions for app features supporting healthy eating, (2) suggestions for reducing food waste, and (3) suggestions for enhancing user experience. This section presents the key themes and subthemes identified under the first category, highlighting graduate students’ perspectives, needs, and expectations for mobile food apps aimed at promoting healthy eating.

### Suggestions for App Features Supporting Healthy Eating

#### Nutrition Knowledge and Awareness

Participants expressed a strong interest in features that can enhance their understanding of nutritional values, including calorie counts, portion sizes, and macronutrient and micronutrient contents. Several noted that while they were aware of general dietary recommendations, they lacked the tools or knowledge to apply them in daily life.

I’d like to see how many calories and how many servings a recipe makes.

[Speaker 10]

I want to know what number of nutritional values my body and my weight need. I want calorie count and portion size count for sure in the app.

[Speaker 7]

I would like to know the food values...calories, protein amount, etc.

[Speaker 9]

I know whether I need to eat more protein or veggies...but I don’t know how to count nutrition info. I like to eat healthier, but I have limited information about healthy eating.

[Speaker 4]

Participants also suggested incorporating guidance on food storage and freshness to support healthier food choices.

Tell me which foods are good for meal planning and what foods are good for immediate consumption.

[Speaker 7]

#### Food Logging and Reminders

Given demanding academic schedules, many graduate students reported frequently skipping meals or forgetting to eat. Participants expressed a desire for gentle reminders or logging tools to help them monitor and reinforce regular eating habits.

Sometimes you just need that nudge...like a check-in. Have you eaten yet?

[Speaker 5]

The doctor told me, I really need to eat breakfast. I need to remind myself.

[Speaker 4]

It would be cool to have something like a logbook or reminder...like your mom asking if you’ve eaten.

[Speaker 5]

#### Personalized Dietary Filters

Participants highlighted the importance of customizing the app based on individual dietary restrictions or preferences. Suggestions included enabling filters for gluten-free, vegetarian, or vegan recipes.

I want vegetarian recipes..., mixed of onion, eggplants and potato, tomato, something like that...

[Speaker 7]

I don’t eat red meat. I think in my belief, chicken is better than meat.

[Speaker 8]

Add gluten-free or vegan preferences to profile to filter recipes.

[Speaker 10]

#### Batch Meal Planning

Batch cooking was a common strategy among students to manage their time more efficiently. Many suggested that a meal planning feature, especially one tailored for weekly preparation, would be highly beneficial.

Most of the time, I prefer to cook at the weekend for all of the days after that. So, I need a healthy weekly plan.

[Speaker 8]

It’s been close to six months that I’ve been scheduling weekly meals...it’s working for me.

[Speaker 1]

I prefer to cook in batches on days that I don’t have work.

[Speaker 7]

#### Simplicity and Quick Recipes

Simplicity and time efficiency were the top priorities of some participants. Students favored apps with quick, simple, and easy-to-follow recipes requiring minimum cooking skills or preparation times.

I want short recipes that are easy to use...not hard cooking.

[Speaker 8]

I’m not into spending...one- or two-hours cooking meals.

[Speaker 1]

### Suggestions for Reducing Food Waste

#### Challenge of Food Waste

Graduate students acknowledged food waste as a frequent challenge, often stemming from overpurchasing, the forgetting of already purchased ingredients in the refrigerator, or a lack of time to cook.

I throw out a lot of food unfortunately, especially veggies, and I hate myself for that.

[Speaker 3]

Sometimes I bought [groceries] thinking I’d have time to cook...then realized I didn’t.

[Speaker 2]

The main waste I deal with is fruits and vegetables that I overbuy and forget about...like getting eight apples but only eating one, so the rest go bad.

[Speaker 5]

To address these challenges, participants offered some recommendations for app features that could help minimize food waste and promote mindful consumption.

#### Smart Recipe Generator Based on Available Ingredients

Participants emphasized the usefulness of a smart recipe feature that generates recipes on the basis of ingredients they already have at home. This would allow them to consume existing food before it spoils.

You give the ingredients, and the app gives you a recipe...that would be great.

[Speaker 3]

I want to find a recipe based on what I have in the fridge.

[Speaker 7]

I want to enter what ingredients I have...and ask the app for recipes based on them.

[Speaker 9]

#### Grocery List Generator With Storage Reminders

Another common suggestion involved integrating a smart grocery list feature, one that is dynamic, saves previous lists, and provides reminders based on items already purchased. Participants noted that food waste occurred often due to overbuying and forgetting what they already had.

It should give me a grocery list according to each meal or all the meals I plan to cook for the next few days.

[Speaker 10]

I want to choose the recipes...and the app recommends what to buy and how much to buy.

[Speaker 3]

Most of the time, my food goes to waste because I overbuy, and I forget.

[Speaker 1]

Save my grocery lists and notify me— ‘Oh, you still have some of this,’ so you can take it off your shopping list.

[Speaker 5]

I found some tomatoes at the back of my refrigerator...and grapes...I totally forgot.

[Speaker 1]

#### Ingredient Substitutions

To avoid food waste due to unavailable ingredients, participants suggested including a substitution feature in the app. This would allow users to complete recipes with the available alternatives, especially when certain cultural ingredients or specialty items are hard to find.

Sometimes you don’t really have that ingredient...alternative choices could avoid waste.

[Speaker 2]

If it’s Chinese food and we can’t find the ingredients, what can we substitute?

[Speaker 3]

### Suggestions for Enhancing User Experience

In addition to functional suggestions related to healthy eating and food waste, participants requested several preferences and expectations for the overall design and usability of the app. These proposals centered on improving clarity, personalization, efficiency, and engagement.

#### Video- and Text-Based Recipes

Participants emphasized the value of having both visual and textual content to accommodate different learning styles and enhance comprehension. While some preferred watching videos for better engagement and understanding of cooking techniques, others found text-based instructions essential for quick references.

Some easy button or colorful button with good illustration...design is important.

[Speaker 8]

I don’t want to replay videos...text version with steps is necessary.

[Speaker 9]

I want to see videos of the food, how to cook them...helps me get engaged better.

[Speaker 1]

If you don’t know what a roux is...you need to see it.

[Speaker 5]

#### Portion Control Feature

Participants expressed a desire for an app that supports flexible portion control options, allowing them to cook for specific durations or numbers of people. This would help them manage meal planning better, reduce waste, and avoid unnecessary leftovers.

I want it to give me the options to cook for one day, two days, etc....and for two people, four people...

[Speaker 1]

It should tell me exact amounts of ingredients, like for four persons.

[Speaker 9]

#### Simplified Onboarding Process

Time constraints were a recurring concern, and participants were discouraged with apps that require long registration processes. They recommended a fast, streamlined onboarding experience to improve user retention and reduce initial barriers.

Not a lengthy process of registration in the app...I don’t have time.

[Speaker 2]

#### Bookmarking and Sharing Recipes

Participants believed that social and organizational features such as bookmarking favorite recipes and sharing recipes with friends were convenient and motivating.

I like to Favorite some recipes, share them with a friend, that would be cool.

[Speaker 5]

#### Accurate Time Estimates

Accurate cooking time estimates were another priority, as students needed realistic expectations to better manage their schedules.

I don’t like when the recipe says 30 minutes cooking time...but it actually takes one hour.

[Speaker 2]

## Discussion

### Principal Findings

This mixed methods study examined graduate students’ dietary behaviors, food waste practices, and preferences for mobile food app features to inform user-centered app design. Survey results revealed that while the majority of participants valued nutrition in food choices, most consumed only 1 serving of vegetables and fruits daily, with over half of the participants eating out daily or almost daily. Nearly 70% (44/63) reported food waste, primarily from leftovers and unused ingredients. A significant association was found between shopping list use and reduced food waste. The follow-up interviews contextualized these patterns, identifying time constraints, limited cooking skills, and overbuying as key barriers. Participants recommended app features such as nutrition tracking, batch meal planning, ingredient-based recipe generators, and smart grocery lists to support healthier eating and reduce waste.

Quantitative results indicated that while nearly half of the participants cooked a few times per week, only a small proportion of participants cooked daily or engaged in structured weekly meal preparation. This finding is aligned with existing research showing that young adults, particularly students, face numerous barriers to healthy eating due to academic pressure, stress, limited time, and low culinary skills as well as financial constraints [35,36]. Qualitative interviews expanded on these patterns, describing how busy schedules contribute to irregular eating patterns and a reliance on convenient, processed foods, a pattern echoed in this study where participants frequently consumed fast food or frozen meals. Additionally, limited intake of fruits and vegetables among participants aligns with broader dietary trends indicating inadequate nutrient consumption among college students [13]. Most participants consumed only 1 serving daily, representing less than 50% of the United States Department of Agriculture (USDA) recommendation of 2.5 cups of vegetables and 2 cups of fruit per day for adults [37]. Nearly 30% reported no daily fruit intake, suggesting substantial gaps in dietary quality that may have long-term health implications. Interviews clarified why this gap existed between students’ intentions and behaviors: while nutrition “mattered,” they lacked the time and skills to translate this value into practice. This pattern suggests that app features promoting vegetable and fruit consumption, such as produce-focused recipes, meal planning tools emphasizing plant-based ingredients, and nutrition tracking, are especially critical for this population.

Participants expressed strong interest in increasing their nutritional awareness, specifically requesting features such as calorie tracking, portion control, and macronutrient breakdowns. These preferences are consistent with literature showing that practical, serving-based nutritional information enhances the appeal and utility of eHealth interventions [38,39]. Students also recommended the inclusion of food logging tools and personalized dietary filters (eg, vegetarian, gluten-free, and vegan), showing their desire for tailored support in maintaining consistent and health-conscious routines. This reflects a broader trend in mHealth design favoring personalized digital interventions that adapt to the health goals and lifestyles of mobile app users.

The combination of survey and interview results also reveals the importance of reminders and nudges. Quantitative data showed irregular cooking habits, and the interviews explained this finding, as participants described how meals were often skipped during busy days. Therefore, participants suggested food logging and gentle reminders that could help address these disruptions. They described how simple prompts, such as reminders to eat or check in on meals, would be helpful in addressing the reality of busy graduate student life, where irregular or skipped meals are common. This is consistent with prior evidence that digital self-monitoring and behavioral nudges are effective in promoting healthy eating routines [40].

Food waste also emerged as another major theme, with nearly 70% (44/63) of participants reporting frequent waste, particularly due to leftovers and unused raw ingredients. This finding is consistent with literature identifying young adults, especially those living alone or with roommates, as major contributors to household food waste [41,42]. Quantitative results linked shopping list use to reduced waste, which was also supported by qualitative insights mentioning that lists could increase organization and prevent overpurchasing. Participants who used shopping lists were less likely to report waste, supporting previous findings that list making encourages healthier food choices and better organization [43,44]. In a previous study, automated list generation features based on meal plans were also recommended to improve app usability and long-term engagement [39].

Moreover, participants proposed additional waste reduction strategies, including smart food recipe generators based on available ingredients, ingredient substitution suggestions, and reminders for unused groceries. These features can increase interest in mobile apps that can go beyond planning to offer real-time decision support, an approach advocated in prior research [45] on behaviorally targeted interventions.

In addition, participants expressed interest in features such as batch meal planning, quick and simple recipes, and a portion control option. These preferences highlight graduate students’ desire for convenience and efficiency in app design, reflecting their need for tools that can adapt to demanding schedules. Previous studies similarly noted time constraints as a barrier to healthy food provision [46,47].

Ease of use and interface design were also important features for participants. Participants expressed a strong preference for apps with intuitive design, efficient onboarding, and multimodal instructional content (eg, video and text). These preferences align with research showing that apps are more likely to be adopted and consistently used if they are visually engaging, easy to navigate, and respect users’ limited time [48,49]. Video tutorials combined with text-based instructions were especially suggested for accommodating different learning styles and improving engagement. Interview participants further emphasized the need for accurate time estimates for meal preparation, as unrealistic expectations can lead to frustration and potential app abandonment.

The following sections present the systematic integration of these findings and their theoretical mapping to behavior change frameworks.

### Integration of Quantitative and Qualitative Findings

The explanatory sequential mixed methods design facilitated the systematic integration of quantitative patterns with qualitative explanations. Qualitative and quantitative findings were merged through a joint display approach, systematically comparing survey patterns with interview themes to generate integrated interpretations. For each major finding, we examined (1) the prevalence and patterns from the quantitative data, (2) the underlying reasons and contextual factors from the qualitative data, and (3) how these combined insights informed app design recommendations.

The survey data identified the occurrence and prevalence of specific behaviors, whereas the interviews illuminated why these patterns emerged and what solutions participants proposed. For example, quantitative findings revealed that 69.8% (44/63) of participants experienced food waste, while interviews uncovered the key mechanisms: overbuying due to poor planning, forgetting of purchased ingredients, and inadequate leftover management. This integration showed that food waste stemmed not merely from a lack of knowledge but from interconnected barriers, including time constraints (opportunity), limited planning skills (capability), and organizational challenges.

Similarly, although 85.7% (54/63) of participants reported valuing nutritional information, interviews explained why this motivation often failed to translate into action: deficits in cooking skills (capability) combined with severe time pressures (opportunity barriers). These integrated insights enabled theoretically grounded, user-centered app recommendations that address the full range of barriers rather than isolated factors alone.

### Mapping Findings to the COM-B Framework

The varied recommendations regarding nutritional support, such as time-saving features, and usability preferences can be systematically understood through the COM-B framework. Capability barriers included limited cooking skills and nutritional knowledge, which students addressed by requesting instructional features such as video tutorials and nutrition tracking. Opportunity barriers centered on time constraints and demanding schedules, leading to recommendations for action planning tools (batch meal planning), prompts and cues (eating reminders), and environmental restructuring (ingredient-based recipe generators, smart grocery lists). While motivation to eat healthily was present (85.7% valued nutrition), it was insufficient without addressing capability and opportunity deficits, highlighting the students’ need for simplified interfaces and personalized dietary filters to sustain engagement. The statistically significant association between shopping list use and reduced food waste (*P*=.008) provides empirical support for action planning as an effective BCT in this population. This mapping demonstrates that effective app design must address multiple COM-B components concurrently, as apps focusing solely on motivation or capability without addressing opportunity barriers are unlikely to achieve sustained behavior change among time-constrained graduate students.

These theory-informed design recommendations provide a foundation for developing interventions that systematically target the multifaceted barriers faced by graduate students in maintaining healthy eating behaviors and reducing food waste.

### Implications

This study contributes meaningfully to the growing body of mHealth research by integrating quantitative behaviors and qualitative preferences among graduate students, a population often overlooked in digital nutrition interventions. The findings demonstrate that graduate students are willing to engage with food-related apps when they are time-saving, informative, and seamlessly integrated into their routines. Key features identified as crucial for adoption included flexible onboarding, clear recipe formats, bookmarking, social sharing capabilities, and adjustable portion sizes.

For app developers and public health professionals, these findings highlight the need to balance evidence-based nutritional guidance with user-centered design principles to ensure long-term engagement and meaningful health outcomes. The systematic mapping of barriers to the COM-B framework and BCTs provides actionable guidance for developing interventions that simultaneously address capability deficits, opportunity barriers, and motivation maintenance.

Future research should build on these insights by developing and testing app prototypes, evaluating long-term usability, engagement, and health outcomes. Translating user-identified features into prioritized, testable requirements using frameworks such as the MoSCoW (Must have, Should have, Could have, Won’t have) method or Kano analysis would strengthen intervention development and enable the systematic evaluation of feature effectiveness with measurable outcomes (eg, vegetable servings per day, grams of food waste per week). Considering the diverse needs of student populations, including cultural backgrounds, dietary preferences, food insecurity, and mental health concerns, future design should prioritize inclusivity and accessibility through cross-sector collaboration.

### Limitations

The sample was limited to a single university in the United States and may not be representative of graduate students at other institutions or in different regions. Additionally, the study relied on convenience sampling through course-based recruitment, which may introduce selection bias. Students enrolled in the 4 targeted courses may differ systematically from the broader graduate student population in terms of academic disciplines, schedules, or other characteristics. Future research should include larger, more diverse samples encompassing students from multiple universities.

The survey measurement of food waste was intentionally broad, assessing presence or absence and primary sources without quantifying amounts, distinguishing avoidable from unavoidable waste, or specifying time horizons. Moreover, interview data relied on recall and self-report rather than objective waste measurement. Future research using food waste diaries, photography-based methods, or actual waste weighing would provide more precise quantification and enable stronger comparisons with the existing literature on food waste.

This study relies entirely on self-reported data for dietary behaviors, cooking frequency, and food waste, which are subject to well-documented biases that may affect data validity. Social desirability bias may have led participants to overreport healthy behaviors (eg, vegetable consumption, meal planning) and underreport less desirable behaviors (eg, eating out, food waste), particularly given their awareness that the study focused on healthy eating and sustainability. Recall bias is another significant concern, as participants were asked to estimate their typical behaviors (eg, daily vegetable servings, weekly cooking frequency) without reference to actual food logs or objective measurements. Research demonstrates that self-reported dietary intake is notoriously unreliable, with individuals often misestimating portion sizes, forgetting eating occasions, and inaccurately recalling consumption patterns [50,51].

Food waste estimates are particularly challenging to measure accurately, as participants may not notice or remember all discarded food, and definitions of “food waste” may vary across individuals. Similarly, cooking frequency relies on subjective interpretation of what constitutes “cooking” versus “meal preparation.” The interview setting may have further influenced disclosure patterns: participants in in-person interviews may have been more susceptible to social desirability bias due to direct interaction with the researcher, while those in online interviews may have felt more comfortable disclosing less favorable behaviors but may have been more distracted or less engaged, potentially affecting response depth and accuracy.

Given these limitations, the behavioral data reported in this study should be interpreted as participants’ perceptions and self-assessments of their dietary patterns rather than objective measurements of actual intake or waste. The primary value of this study lies not in precise quantification of dietary behaviors but in understanding students’ experienced barriers, perceived needs, and preferences for app features—insights that remain valid even if absolute frequencies are subject to reporting bias. Future research should incorporate objective measures such as food photography, actual grocery receipts, weigh-and-measure food waste protocols, or ecological momentary assessment to validate self-reported patterns and provide more accurate behavioral data.

The sample size of 63, while adequate for descriptive analysis and the exploratory mixed methods design, provided moderate statistical power (0.78) for the single chi-square test conducted. Although this approaches conventional power thresholds and the observed association was statistically significant (*P*=.008), larger samples would provide greater power to detect smaller effect sizes and enable the exploration of additional potential associations. The focus on a single inferential test avoided issues with multiple comparisons and type I error inflation.

Data collection occurred during November and December 2024, which may have influenced participants’ dietary and shopping behaviors. This period coincides with the end of the fall semester, when academic pressures and final examinations peak, potentially affecting cooking frequency, meal planning, and reliance on convenience foods. These seasonal factors should be considered when interpreting the findings, and future research conducted across multiple time points throughout the academic year would provide a more comprehensive understanding of graduate students’ typical dietary patterns and food waste behaviors.

This study did not systematically assess contextual environmental factors such as participants’ access to kitchen facilities, proximity to grocery stores, or availability of on-campus dining options, all of which may influence cooking frequency, shopping behaviors, and food waste patterns. Additionally, food insecurity status was not assessed although it may moderate both diet quality and waste behaviors. Future research should examine how the campus food environment and food security status shape graduate students’ dietary practices and app feature preferences.

### Conclusion

This study demonstrates that effective mobile food apps for graduate students must integrate evidence-based nutritional guidance with user-centered design principles that accommodate time constraints, varying cooking skills, and organizational challenges. While graduate students value healthy eating and wish to reduce food waste, practical barriers prevent them from translating intentions into behaviors.

By mapping findings to the COM-B framework and BCTs, this study identified graduate students’ needs for app features that simultaneously address capability deficits (eg, nutrition knowledge and cooking skills), opportunity barriers (eg, time constraints and planning challenges), and motivation maintenance (eg, personalization and simplicity). The findings underscore 3 key design principles: first, food apps must function as comprehensive support systems offering personalized dietary guidance, proactive reminders, and waste prevention tools, not merely recipe repositories. Second, adoption and sustained engagement depend heavily on simplicity, multimodal content delivery, and realistic time expectations. Third, features simultaneously addressing both healthy eating and food waste are likely to have synergistic effects, as organized meal planning supports both nutritional goals and resource conservation.

These insights may inform mHealth tool development for other populations, apart from graduate students, facing similar time pressures and organizational challenges, including working adults, parents, and health care professionals. Future work should focus on developing and rigorously testing prototypes that incorporate these user-identified features, evaluating their real-world effectiveness in promoting sustained dietary behavior change and food waste reduction. Cross-sector collaboration involving app developers, nutritionists, behavioral scientists, and student populations will be essential for creating scalable, inclusive digital health solutions that translate user preferences into measurable health and environmental outcomes.

## Supplementary material

10.2196/84576 Multimedia Appendix 1 Survey questions.

## Abbreviations

BCT: behavior change technique
COM-B: capability, opportunity, and motivation-behavior
mHealth: mobile health
MoSCoW: Must have, Should have, Could have, Won’t have
USDA: United States Department of Agriculture
