# Supplementary material for: Understanding Graduate Students’ Perspectives on Food Apps to Inform User-Centered Design: Explanatory Sequential Mixed Methods Survey Study
Source: JMIR Form Res. 2026 Mar 27;10:e84576. doi: 10.2196/84576 (PMC13026433; doi:10.2196/84576)
Supplement: Multimedia Appendix 1 [file formative-v10-e84576-s001.docx]

**Survey Introduction**

Dear Participant,

Thank you for participating in this research study. My name is Mehrnoush Mokhtarnejad. I am a doctoral student in the Library and Information Science, School of Information Science at the University of South Carolina. We are investigating the food habits and preferences of graduate students at the University of South Carolina to develop a user-centric mobile application that encourages healthy eating and reduces food waste. Your responses will help us understand how to design a food application that meets your needs, saves you time, and promotes a healthier lifestyle.

The survey will take approximately 5 minutes to complete, and your responses will be anonymous. If you have any questions or would like to participate in a follow-up interview, please provide your contact information at the end of the survey.

Participation in this survey is voluntary, and you may withdraw at any time. If you have any questions or concerns about the research study, please email me at mokhtarm@email.sc.edu

- **Demographics:**

**1.** What is your age?

- 22 to 32

- 33 to 44

- 45 to 55

- Over 55

**2.** What is your gender?

Short answer text _______

**3.** How would you describe your race/ethnicity? (Select all that apply)

White (non-Hispanic)

Black or African American (Non-Hispanic)

Hispanic/Latino(a)

Asian/ (AAPI)

Middle Eastern or North African (MENA)

Multiracial/Other (Please specify): _______

**4.** What smartphone operating system do you use?

- Android

- iOS

- Other: _______

**5.** What program are you currently enrolled in?

- PhD Program

- Master’s Program

- MFA Program

- Other: _______

- **Living and Working Situation:**

**6.** What is your current living situation?

- Living alone

- Living with roommates

- Living with partner or family

**7.** What is your occupation? (Select all that apply).

Full-time student

Part-time student

In-person job

Remote work

Hybrid work (combination of in-person and remote)

- **Cooking and Food Habits:**

**8.** How often do you cook?

- Rarely or never

- A few times a month

- I meal prep for the entire week

- A few times a week

- Daily

**9.** On average, how long does it take you to prepare a meal?

- 15 minutes or less

- 16 to 30 minutes

- 31 to 60 minutes

- More than an hour

**10.** How much does the nutritional value of ingredients influence your food choices?

- A great deal

- Quite a bit

- Somewhat

- Very little

- Not at all

**11.** How often do you use fast food, frozen, or canned food?

- Never

- A few times a month

- 1–2 times a week

- 3–5 times a week

- Every day

**12.** How would you describe your diet?

- balanced meals, mostly whole foods, minimal processed foods (Very healthy)

- generally balanced, occasional indulgences (Mostly healthy)

- Mix of healthy and unhealthy foods (Moderately healthy)

- Frequent processed or fast foods, but some healthy choices (Somewhat unhealthy)

- Mostly processed or fast foods, little focus on nutrition (Unhealthy)

**13.** How many servings of vegetables do you eat per day?

None

One

Two

Three or more

**14.** How many servings of fruit do you eat per day?

None

One

Two

Three or more

- **Food Waste:**

**15.** Have you ever experienced issues with food waste?

- Yes

- No

**16.** If yes, what is the primary source of your food waste?

- Raw ingredients

- Leftover food

- Other (Please specify): _______

- **Food Recipe and Application Usage:**

**17.** How often do you search for food recipes online (e.g., YouTube, recipe apps)?

- Always

- Sometimes

- Rarely

- Never

**18.** What type of recipes do you mainly search for? (Select all that apply)

Everyday meals (e.g., breakfast, lunch, dinner)

Special or festive dishes (e.g., holiday meals, party foods)

International or ethnic cuisine

Quick and easy recipes (e.g., fast food, simple meals)

Healthy options (e.g., salads, low-calorie dishes)

Sauces and dressings

Beverages (e.g., special drinks, smoothies)

Meal prep and batch cooking (e.g., recipes for preparing meals in advance)

Vegetarian or vegan recipes

Gluten-free or allergen-friendly recipes

**19.** Have you ever used a mobile food app to prepare a meal?

- Yes

- No

**20.** If yes, please name the app(s) you’ve used: _______

**21.** How would you describe the food apps or recipe websites you use? (Select all that apply)

Clear and easy to use

Confusing or difficult to navigate

Helpful and informative

Misleading or inaccurate

Time-saving

Overly complicated

User-friendly with step-by-step guidance

Lacking important details or features

- **Grocery Shopping and Planning:**

**22.** How often do you plan meals before going grocery shopping?

- Always

- Sometimes

- Rarely

- Never

**23.** Do you use a shopping list when you go grocery shopping?

- Yes

- No

**24.** How often do you grocery shop?

- Daily

- Weekly

- Bi-weekly

- Monthly

- Other: _______

**25.** How confident are you in planning meals before grocery shopping?

(Scale 1–5)

**26.** How important is it for you to plan meals before grocery shopping?

(Scale 1–5)

**27.** How often do you eat out?

- Never

- Rarely (1–2 times a month)

- Occasionally (1–2 times a week)

- Frequently (3–5 times a week)

- Daily or almost daily

- **Interest in Interview:**

**28.** If you are willing to participate in a follow-up interview, please provide your email address:

- Email: _______
